# Management of sepsis in Indian ICUs: Indian data from the MOSAICS study

**DOI:** 10.1186/cc11777

**Published:** 2012-11-14

**Authors:** J Divatia

**Affiliations:** 1 Tata Memorial Hospital, Mumbai, India

## Background

Indian data from the Management of Sepsis in Asia's Intensive Care Units (MOSAICS) study are presented. The primary objective of the MOSAICS study was to assess the compliance of Asian ICUs and hospitals to the Surviving Sepsis Campaign's 6-hour resuscitation bundle and 24-hour management bundle.

## Methods

This was prospective cohort study of 162 adult patients with severe sepsis who were admitted to 17 ICUs in India in July 2009. Patients' baseline characteristics, the achievement of targets within the resuscitation and management bundles, and outcome data were recorded. Main outcome measures were compliance to the Surviving Sepsis Campaign's resuscitation and management bundles. Secondary objectives were to evaluate the impact of compliance on mortality.

## Results

The mean age of patients was 53.3 ± 17 years. The mean APACHE II SCORE was 21.9 ± 8.4, and was significantly higher in nonsurvivors (26.3 ± 8.5) than survivors (19.2 ± 7.3). Hospital mortality was 38.3% (62/162). Achievement rates for the bundle targets were: lactate measurement, 40.1% (65/162); blood cultures, 52.4% (85/162); broad-spectrum antibiotics, 62.3% (101/162); fluids ± vasopressors, 84.4% (103/122); central venous pressure, 49.6% (56/113); central or mixed venous oxygen saturation, 16.8% (19/113); low-dose steroids, 52.4% (55/105); glucose control, 27.2% (44/162); lung-protective ventilation, 22.1% (15/68). Compliance rates for the entire resuscitation and management bundles were 6.8% (11/162) and 8% (13/162) respectively. There was a trend to improving survival with bundle compliance. Compliance with the resuscitation and management bundles was associated with mortality rates of 18.2% and 19%, respectively, while mortality rates with noncompliance were 39.7% and 41.1%. Of the resuscitation bundle elements, achieving targets for fluid resuscitation and vasopressors was associated with survival, but this disappeared when adjusted for APACHE II score. Of the elements in the management bundle, achieving glucose target and low tidal volume ventilation in patients with acute lung injury as well as the APACHE II score were independently associated with survival.

## Conclusion

While mortality from severe sepsis is high, compliance to the resuscitation and management bundles is generally poor in ICUs in India.

